# In-person vs mobile app facilitated life skills education to improve the mental health of internally displaced persons in Nigeria: protocol for the RESETTLE-IDPs cluster randomized hybrid type 2 effectiveness-implementation trial

**DOI:** 10.1186/s12913-024-11762-x

**Published:** 2024-10-22

**Authors:** Ejemai Eboreime, Chisom Obi-Jeff, Rita Orji, Tunde M Ojo, Ihoghosa Iyamu, Bala I Harri, Jidda M Said, Funmilayo Oguntimehin, Abdulrahman Ibrahim, Omolayo Anjorin, Andem Effiong Etim Duke, Umar Baba Musami, Linda Liebenberg, Raquel Crider, Lydia Wagami, Asmau MC Dahiru, Jesse C. Uneke, Sanni Yaya, Vincent IO Agyapong

**Affiliations:** 1 Department of Psychiatry, Faculty of Medicine, Dalhousie University, Halifax, NS Canada; 2 Brooks Insights, Abuja, Nigeria; 3 London School of Hygiene and Tropical Medicine, London, UK; 4 Faculty of Computer Science, Dalhousie University, Halifax, NS Canada; 5 Department of Public Health, Federal Ministry of Health and Social Welfare, Abuja, Nigeria; 6 Department of Psychiatry, University of Abuja, Abuja, Nigeria; 7 British Columbia Centre for Disease Control, Vancouver, BC Canada; 8 School of Population and Public Health, University of British Columbia, Vancouver, BC Canada; 9 Department of Health Planning, Research and Statistics, Federal Ministry of Health, Abuja, Nigeria; 10 Department of Mental health, University of Maiduguri Teaching hospital, Maiduguri, Nigeria; 11 Mental Health Department, University of Maiduguri, Maiduguri, Nigeria; 12 Federal Neuropsychiatric Hospital, Maiduguri, Nigeria; 13 Department of Statistics, Food Ingredient and Health Research Institute, Naalehu, HI USA; 14 National Emergency Management Agency, Abuja, Nigeria; 15 African Institute for Health Policy & Health Systems II, David Umahi Federal University of Health Sciences, Uburu, Nigeria; 16 Imperial College London, The George Institute for Global, London, UK; 17 QEII Health Sciences Centre, 5909 Veterans’ Memorial Lane, 8th Floor Abbie J. Lane Memorial Building, Halifax, NS B3H 2E2 Canada

**Keywords:** Internally displaced persons, Mental health, Life skills education, Armed conflict, Sexual violence, MHealth, WhatsApp, Digital Health, Forced displacement, Nigeria

## Abstract

**Background:**

Internally displaced persons (IDPs) in Nigeria face a high burden of mental health disorders, with limited access to evidence-based, culturally relevant interventions. Life skills education (LSE) is a promising approach to promote mental health and psychosocial well-being in humanitarian settings. This study aims to evaluate the effectiveness and implementation of a culturally adapted LSE program delivered through in-person and mobile platforms among IDPs in Northern Nigeria.

**Methods:**

This cluster-randomized hybrid type 2 effectiveness-implementation trial will be conducted in 20 IDP camps or host communities in Maiduguri, Nigeria. Sites will be randomly assigned to receive a 12-week LSE program delivered either through in-person peer support groups or WhatsApp-facilitated mobile groups. The study will recruit 500 participants aged 13 years and older. Intervention effectiveness outcomes include the primary outcome of change in post-traumatic stress disorder (PTSD) symptoms assessed using the PCL-5 scale, and secondary outcomes of depression, anxiety, well-being, and life skills acquisition. Implementation outcomes will be assessed using the Acceptability of Intervention Measure (AIM), Intervention Appropriateness Measure (IAM), and Feasibility of Intervention Measure (FIM). Both sets of outcomes will be compared between the in-person and mobile delivery groups. Quantitative data will be analyzed using mixed-effects linear regression models, while qualitative data will be examined through reflexive thematic analysis. The study will be guided by the Reach-Effectiveness-Adoption-Implementation-Maintenance (RE-AIM) framework.

**Discussion:**

The RESETTLE-IDPs study addresses key gaps in the evidence base on mental health interventions for conflict-affected populations. It focuses on underserved IDP populations, evaluates the comparative effectiveness of in-person and mobile-delivered LSE, and incorporates implementation science frameworks to assess contextual factors influencing adoption, fidelity, and sustainability. The study employs a community-based participatory approach to enhance cultural relevance, acceptability, and ownership. Findings will inform the development and scale-up of evidence-based, sustainable mental health interventions for IDPs in Nigeria and other humanitarian contexts.

**Trial sponsor:**

Dalhousie University, 6299 South St, Halifax, NS B3H 4R2, Canada.

**Trial registration:**

ClinicalTrials.gov, NCT06412679 Registered 15 May 2024.

**Supplementary Information:**

The online version contains supplementary material available at 10.1186/s12913-024-11762-x.

## Background

Nigeria is facing a humanitarian crisis, with over 3.2 million people internally displaced due to armed conflicts, communal violence, and natural or man-made disasters exacerbated by climate change [1, 2]. As the United Nations Office for the Coordination of Humanitarian Affairs (OCHA) explains, “People become internally displaced when they are forced to leave their homes due to conflict, violence, human rights violations, natural hazards, or other crises within the borders of their country. This can include situations where people move voluntarily to seek safety or to access essential services” [3]. The Boko Haram insurgency and farmer-herder clashes have been major drivers of forced displacement in Nigeria, particularly in the northern regions [4]. This mass displacement has led to a surge of internally displaced persons (IDPs) who live within the borders of the country, in overcrowded, under-resourced camps and host communities, where they face numerous challenges to their physical and mental well-being [5–7].

Mental health disorders are alarmingly prevalent among Nigerian IDPs compared to the general population. A recent study found that 75% of IDPs affected by the Boko Haram conflict experienced clinically significant psychological distress [8]. The prevalence of symptoms of post-traumatic stress disorder (PTSD) ranges from 75 to 94% among IDPs, while symptoms of depression and anxiety were experienced by 60–98% and 66–99%, respectively [8–10]. These rates far exceed the national averages of 20–30% for mental health disorders in the general population [11]. The mental health burden is also high among internally displaced children and adolescents, with up to 67% experiencing PTSD and 54% showing symptoms of depression [12–14]. Considering that the onset of most mental illnesses occurs between the ages of 12 and 25 years [15]. Traumatic experiences such as conflicts, famine, and forced displacement can have significant immediate and long-term impacts on these developing minds, including acute stress reactions, PTSD, chronic depression, substance abuse, and difficulties in social and occupational functioning [16]. These experiences during critical developmental stages can shape an individual’s mental health trajectory well into adulthood.

Women and girls in IDP camps are disproportionately impacted by sexual exploitation, trafficking, forced marriage, and other forms of gender-based violence (GBV) [17]. A study in Northeast Nigeria found that over one-third of displaced women experienced sexual violence, while a fifth reported physical violence. However, only a fraction sought care due to the limited availability of GBV services and fear of stigma [17]. Displacement also disrupts access to education, livelihoods, and social support systems, further compounding the risks to mental health and psychosocial well-being.

Despite the immense needs, there is a dearth of evidence-based, culturally-adapted interventions to address the mental health crisis among IDPs in Nigeria and other humanitarian settings. Most existing services focus on short-term relief aid rather than psychosocial supports and resilience-building resources that would enable IDPs sustainably support themselves move towards mental health and well-being [18]. Conventional facility-based mental healthcare is often inaccessible or inadequate to meet the complex needs of IDP populations due to inadequate mental healthcare workforce, with less than 300 psychiatrists for a population of over 200 million [19–21].

Life skills education (LSE) has emerged as a promising approach to promoting mental health and well-being among conflict-affected populations [22–25]. The World Health Organization (WHO) defines life skills as ‘*a set of abilities that enable individuals to effectively handle the challenges and demands of daily life*’ [26]. LSE programs aim to equip individuals with cognitive, behavioural, and interpersonal skills to effectively cope with stressors, navigate challenges, and make healthy decisions [27, 28]. Studies have shown that LSE interventions can significantly reduce symptoms of depression, anxiety, PTSD, and aggression while improving self-esteem, social connectedness, and problem-solving among youth in humanitarian settings. For instance, one study of young adults in Iran found that, following 2 months of LSE, the mean scores of poor general health decreased by 22 (SD = 18.15, t = 11.2), anxiety decreased by 6.17 (SD = 4.2, t = 8.94) (*P* < 0.01,), social function deficit decreased by 4 (SD = 5.5, t = 6.35), and depression decreased by 7.3 (SD = 3, t = 6.6) [29]. Another clinical trial investigating the effectiveness of LSE on the mental health of middle school students found that LSE had a significant effect on four variables of mental health (stress, violence, addiction, sensation-seeking) (Eta-2 = 0.245; *p* < 0.001) [30]. Additionally, a clinical trial demonstrated the effectiveness of LSE in enhancing food resource management ability, health, and food security status among individuals seeking financial aid to avoid homelessness [29]. Another review found small to medium effect sizes for mental health outcomes and large effect sizes for relevant life skills across various adolescent health areas, including mental health. The effect sizes were categorized as small, medium, or large based on the magnitude of the standardized mean difference (SMD) calculated for the outcomes analyzed. Specifically, LSE interventions were effective in reducing symptoms of anger (SMD) = 1.234), improving life skills (SMD = 0.755) and functioning (SMD = 0.491), and decreasing PTSD (SMD = 0.327), depression and anxiety (SMD = 0.305). Positively correlated with the following life skills: interventions that targeted parent-child interactions (β = 0.557, *p* < 0.05), the assessment of interpersonal relations (β = 0.204, *p* < 0.05), and stress management (β = 0.216, *p* < 0.05) [31].

Further, most LSE interventions targeted schools (78%), communities (10%) and refugee camps (8%) and were delivered by facilitators such as teachers and specialist providers, and most were focused on vulnerable groups [31]. This highlights the broad scope and target populations of these interventions, as well as the utilizing on training people for their implementation.

Regarding LSE content, 93.3% of articles endorsed behavioral skills, followed by interpersonal skills (86.7%), and cognitive skills (66.7%). Common delivery strategies were individually (55.6%) or in groups (44.4%). None was found using digital technology [31]. Thus, there is evidence that LSE can help displaced persons develop a sense of purpose, increase their self-esteem, and improve their relationships with others, all of which contribute to better mental wellbeing, resilience building and social re-integration. However, the evidence base for LSE among IDPs in Nigeria is limited, and the comparative effectiveness of different delivery models remains unknown. In addition, mental health and psychosocial support services (MHPSS) in Nigeria are not tailored to the unique needs of IDPs due to the limited involvement of the end-users, affecting the acceptability and successful implementation [32, 33]. Empowering IDPs to attain the highest standard of mental health and wellbeing can be achieved through a community-partnered participatory approach to implementation [34]. A community-partnered participatory approach is a collaborative and structured engagement of service users (community members), providers, and local stakeholders in planning and implementing targeted solutions specific to their needs [35–37]. This approach has improved mental health-related quality of life and service use in under-resourced communities [38, 39].

Digital technologies offer new opportunities to expand access to mental health support for hard-to-reach IDP populations [40–42]. Mobile phones are increasingly available and utilized among displaced communities in Nigeria. A recent study found that WhatsApp was the most used social media platform among IDPs in Borno State [43]. Harnessing mobile platforms like WhatsApp to deliver LSE content and facilitate peer support groups could help overcome barriers of stigma, insecurity, and limited resources that hinder in-person participation. However, the feasibility, acceptability, and effectiveness of Mobile-based LSE compared to in-person formats have not been rigorously evaluated in humanitarian contexts.

To address these gaps, we propose the Rebuilding Emotional Stability and Strength Through Therapeutic and Life-Skills Education for Internally Displaced Persons (RESETTLE-IDPs) study, a cluster-randomized type 2 effectiveness-implementation trial that aims to:

1. Co-design and implement a culturally adapted LSE intervention curriculum with IDP communities and local stakeholders in Northern Nigeria;
2. Evaluate the effectiveness of a culturally adapted LSE intervention in improving mental health and psychosocial outcomes among IDPs;
3. Compare the implementation outcomes of in-person vs. Mobile (WhatsApp)facilitated delivery models for the LSE intervention.
4. Assess the contextual factors, including social, economic, and cultural factors and mechanisms influencing the adoption, fidelity, and sustainability of the LSE intervention in IDP settings; and.
5. Engage stakeholders to co-design strategies for scaling up and integrating LSE within existing humanitarian services in Nigeria and beyond.

## Methods and design

**Fig. 1:**
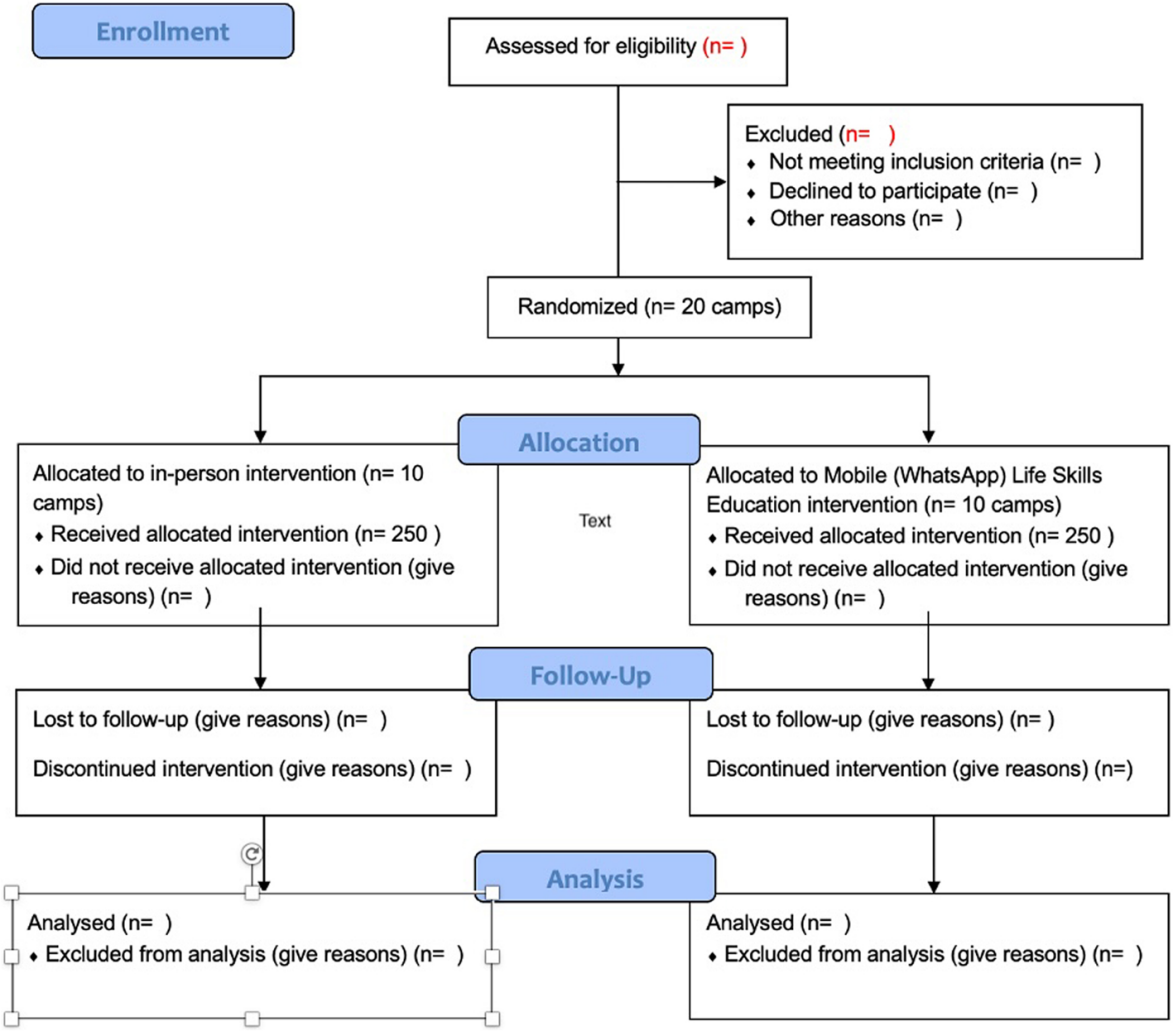
CONSORT flow diagram

This paper was guided by the Consolidated Standards of Reporting Trials (CONSORT) (Fig. 1) and the Standards for Reporting Implementation Studies (StaRI) Checklists (Additional Files 1 & 2).

### Study design

The RESETTLE-IDPs study employs a cluster-ized type 2 hybrid effectiveness-implementation trial design [44]. This design allows for the simultaneous evaluation of the clinical effectiveness of the LSE intervention on mental health outcomes and the assessment of implementation outcomes to inform potential scale-up and sustainability [45]. The design will also allow the comparison of the in-person and the mobile arm.

The study will be conducted in two phases using a community-partnered participatory approach :

- Phase (1) a formative phase to culturally adapt the LSE intervention and engage stakeholders; and.
- Phase (2) an intervention phase to evaluate the effectiveness and implementation of the LSE program delivered through in-person peer support groups or Mobile -facilitated peer groups.

### Study setting and participants

The study will be conducted in IDP host communities in Maiduguri, the capital of Borno State in Northeast Nigeria. Since the emergence of the Boko Haram conflict in 2009, Maiduguri has become the hub of IDPs and hosts the highest proportion of IDPs in the country, 76%. An estimated 49% of these IDPs reside in 237 camp/camp-like settings, and 51% are in host communities [4]. The IDP host communities will be our unit of randomizing, using a computer generation. Selection criteria for our two study sites include population size, non-existence of MHPSS, network availability, and the presence of local partners and infrastructure to support the research.

The target population includes IDPs 13 years and older who have been displaced for at least three months. Participants will be recruited through a combination of purposive sampling strategies, with the assistance of camp leaders, community mobilizers, and humanitarian organizations in those sites, given the intervention delivery approach: in-person and mobile arm group. Inclusion criteria are: (1) resident in IDP camp for 6 months; (2) aged 18 years or older; (3) speaks English or Hausa; (4) having access to a mobile phone or being willing to share one (for the Mobile, i.e., WhatsApp, arm); and (5) providing informed consent/assent. It is important to note that while device sharing is allowed for participation in the Mobile arm, it may pose potential risks to participants’ privacy and confidentiality. Measures will be taken to mitigate these risks, such as providing guidance on secure device sharing practices and ensuring that shared devices have appropriate security features enabled.

Exclusion criteria include (1) the presence of mental illness, cognitive impairment, or substance abuse that would hinder meaningful participation following the baseline assessment; (2) imminent risk of suicide or violence requiring immediate referral; and (3) unwillingness to participate in the study or complete study assessments.

### Sample size and power analysis

The target sample size of 500 participants (250 per arm) was calculated based on the primary outcome of change in PTSD symptoms, as measured by the PCL-5. The sample size was determined to detect a minimum effect size (Cohen’s d) of 0.4, which represents a clinically meaningful difference in PTSD symptoms between the two intervention arms. This effect size is consistent with previous studies of psychosocial interventions for conflict-affected populations [46]. The power calculation assumed a two-sided significance level of 0.05, a power of 80%, and an intra cluster correlation coefficient (ICC) of 0.05 to account for the clustering of participants within IDP camps or communities. The ICC was estimated based on previous cluster-randomized trials of mental health interventions in humanitarian settings [47]. To account for potential attrition, the sample size was inflated by 15%, based on an anticipated dropout rate of 10–15% over the 12-week intervention period. This attrition rate is consistent with previous longitudinal studies of IDPs and takes into consideration the potential challenges of retention in the context of ongoing displacement and insecurity [48].

Based on these parameters, a total of 20 clusters (10 per arm) with an average of 25 participants per cluster (500 participants in total) will provide sufficient power to detect a clinically meaningful difference in PTSD symptoms between the in-person and mobile-delivered LSE interventions, while accounting for clustering and attrition.

### The adapted evidence-based intervention

The LSE intervention will consist of a culturally adapted curriculum covering core life skills topics such as stress management, communication, problem-solving, goal setting, health and hygiene, safety and protection, and social support. The curriculum will be developed through a participatory process involving IDP men and women, community leaders, mental health experts, and humanitarian actors, drawing on existing evidence-based resources, particularly the UNICEF Comprehensive life skills framework [49].

**Fig. 2:**
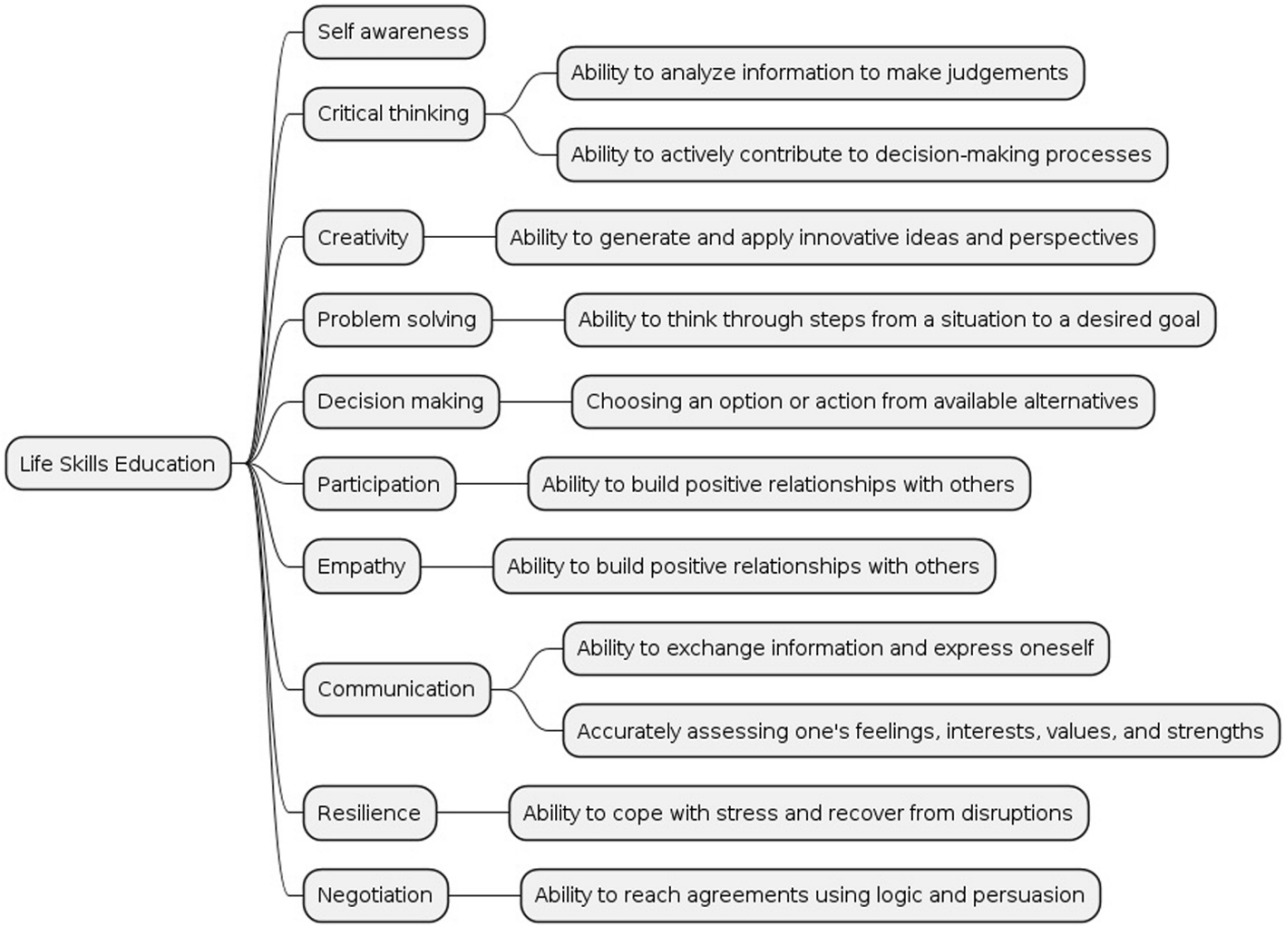
The life skills education scope

Agencies of the United Nations list the ten core life skill strategies and techniques as: problem solving, critical thinking, effective communication skills, decision-making, creative thinking, interpersonal relationship skills, self-awareness building skills, empathy, and coping with stress and emotions [50] (Fig. 2). These skills will be categorized into an initial group of skills that contribute to the four outcome areas of life-long learning, employability and entrepreneurship, personal empowerment, and active citizenship [51]. These transferable skills are building also helps prevent aggressive and conflict inducing behaviour later in life and is essential to any post-crisis reconciliation, social cohesion and longstanding peace [51].

In the in-person arm, participants will attend weekly 120-minute peer support group sessions facilitated by trained IDP men and women and supervised by local mental health workers. The groups will be gender- and age-segregated (13–15, 16–19, and 18 + years) to create safe spaces for open discussion and skills practice. Sessions will employ interactive and experiential learning techniques such as role-plays, storytelling, games, and art-based activities to enhance engagement and retention.

In the mobile arm (i.e. WhatsApp), participants will join gender- and age-specific mobile groups moderated by trained facilitators. The groups will receive weekly LSE content and exercises through a combination of messages, voice notes, images, and live audio sessions. Participants will be encouraged to share reflections, ask questions, and provide peer support through the WhatsApp chat. Facilitators will stimulate discussion, offer feedback, and monitor participant safety and engagement.

Both arms will receive the LSE intervention for a duration of 12 weeks, with an average of 10–12 participants per peer support group. Fidelity to the intervention will be assessed through structured observations, attendance records, and facilitator logs [52]. Participants will receive the equivalent of $20 US and be provided with snacks during the intervention. Participants will also have access to referral information for additional mental health and social services as needed.

### Theory of change

**Fig. 3:**
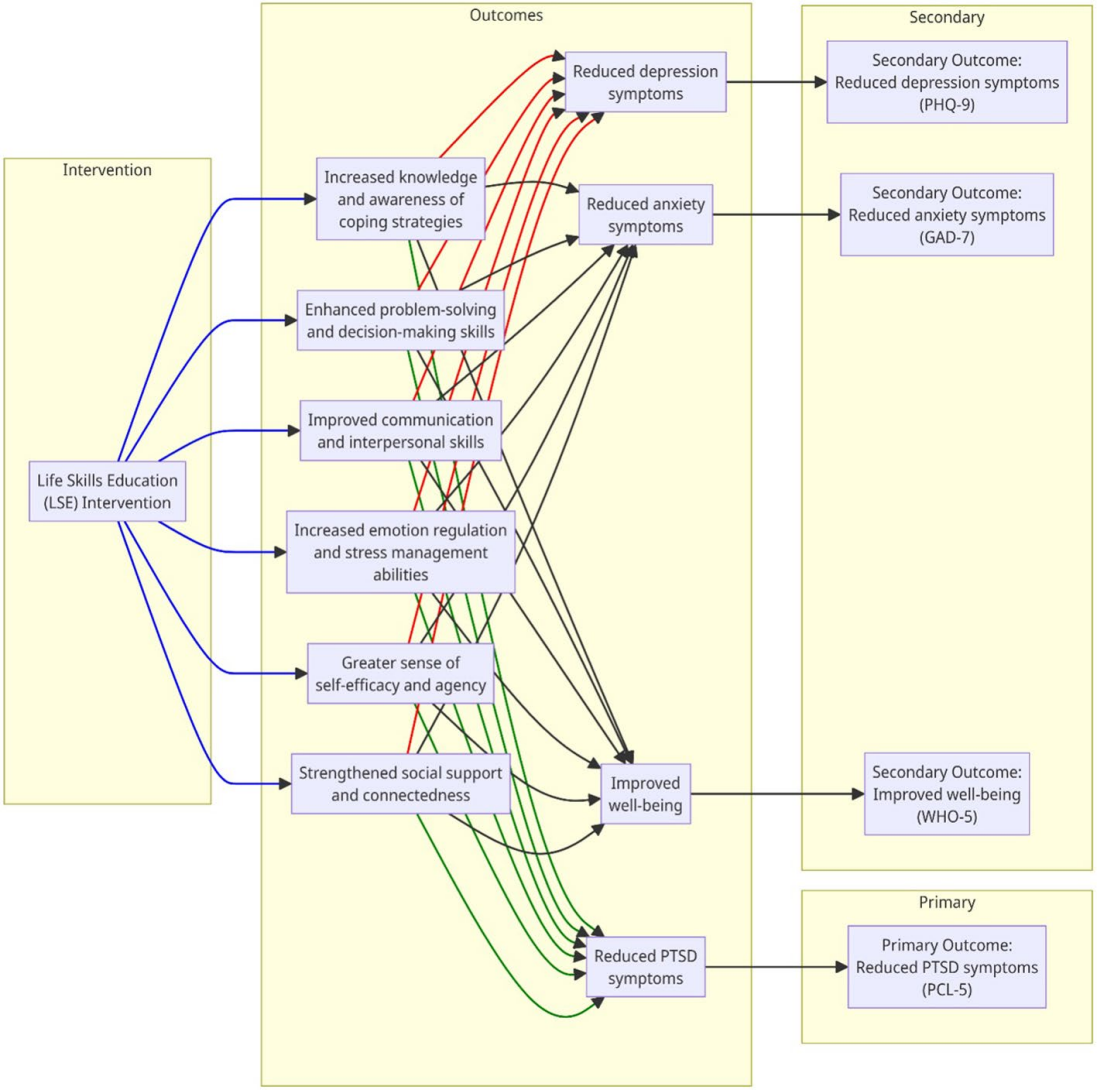
The RESETTLE-IDPs theory of change model

The RESETTLE-IDPs study is guided by a theory of change that posits a pathway from the LSE intervention to improved mental health and well-being outcomes among IDPs (Fig. 3). The LSE intervention is hypothesized to lead to a series of proximal outcomes, including:

1. Increased knowledge and awareness of coping strategies.
2. Enhanced problem-solving and decision-making skills.
3. Improved communication and interpersonal skills.
4. Increased emotion regulation and stress management abilities.
5. Greater sense of self-efficacy and agency.
6. Strengthened social support and connectedness.

These proximal outcomes are expected to mediate the effect of the LSE intervention on the primary outcome of reduced PTSD symptoms, as well as the secondary outcomes of reduced depression and anxiety symptoms and improved well-being. The theory of change also recognizes the potential moderating effects of individual, social, and contextual factors on intervention effectiveness and implementation.

By articulating the hypothesized causal pathways and mechanisms of change, the theory of change provides a conceptual framework for designing the intervention, selecting measures, and analyzing results. It also helps to identify key constructs and processes to be assessed in the process evaluation to elucidate how and why the intervention works in this context. The theory of change will be refined based on study findings and stakeholder input to inform future implementations and adaptations of the LSE intervention for IDPs.

### Outcomes and measures

The study outcomes will be guided by the Reach-Effectiveness-Adoption-Implementation-Maintenance (RE-AIM) framework [53]. Reach will assess the number, proportion, and representativeness of IDP men and women participating in the LSE intervention. Effectiveness will evaluate the intervention’s impact on primary and secondary clinical health outcomes. Adoption will determine the uptake of the LSE intervention among IDP camps and humanitarian organizations. Implementation will examine the fidelity, consistency, and cost of delivering the intervention. Finally, maintenance will evaluate the long-term sustainability using camp reports and LSE intervention within organizational and state programs (Table 1). To maximize the program’s impact, we will assess both its clinical effectiveness and implementation processes. By comprehensively assessing these dimensions, we aim to optimize the intervention’s impact and inform future implementation efforts.

**Table 1:** Outcome measures defined by the RE-AIM framework

RE-AIM Outcome	Operational Definition	Measures
**Reach**	The absolute number, proportion, and representativeness of IDP men and women who participate in the LSE intervention.	- Number and percentage of eligible participants who enroll in the study - Demographic and clinical characteristics of participants compared to the target population
**Effectiveness**	The impact of the LSE intervention on primary and secondary mental health outcomes among IDP youth and women.	- PTSD symptoms (PCL-5) - Depression symptoms (PHQ-9) - Anxiety symptoms (GAD-7) - Well-being (WHO-5) - Life skills acquisition (locally developed measure)
**Adoption**	The absolute number, proportion, and representativeness of IDP camps and humanitarian organizations that agree to deliver the LSE intervention.	- Number and percentage of IDP camps that participate in the study - Number and types of humanitarian organizations that support the intervention
**Implementation**	The fidelity, consistency, and cost of delivering the LSE intervention as intended in real-world IDP settings.	- Adherence to the LSE curriculum and delivery protocol (facilitator logs, observations) - Dosage of intervention received by participants (attendance records) - Quality of intervention delivery (participant ratings, facilitator competence) - Adaptations made to the intervention during implementation (facilitator logs, interviews)
**Maintenance**	The extent to which the LSE intervention becomes institutionalized or part of routine humanitarian programming for IDPs over time.	- Continuation of the LSE intervention beyond the study period (camp reports, observations) - Integration of the LSE intervention into existing humanitarian services (organizational policies, budgets) - Sustained effects of the intervention on mental health outcomes at 6 and 12-month follow-up (PCL-5, PHQ-9, GAD-7, WHO-5) - Perceptions of the intervention’s sustainability and scalability (stakeholder interviews)

Clinical outcomes will focus on primarily the intervention effectiveness in in changing PTSD symptoms from baseline to post-intervention (12 weeks), as measured by the Post-Traumatic Stress Disorder Checklist for DSM-5 (PCL-5) [54]. The PCL-5 is a 20-item self-report measure that assesses the presence and severity of PTSD symptoms on a 5-point Likert scale, with higher scores indicating greater symptom severity. The measure has been validated in several cultural contexts and shows good psychometric properties and will be validated to the local Hausa Language [55]. Secondary outcomes include changes in depression symptoms (Patient Health Questionnaire-9), anxiety symptoms (Generalized Anxiety Disorder-7), well-being (WHO-5), and life skills acquisition (locally developed measure) [56–58].

Implementation outcomes will assess the acceptability, appropriateness, fidelity, and feasibility of the intervention. To gain a deeper understanding of these factors, a qualitative process evaluation will be conducted throughout the study. This evaluation will explore factors influencing program delivery, participant experiences, and program outcomes, including barriers and facilitators to implementation. Data will be collected through in-depth interviews with program facilitators, key stakeholders, and participants, as well as focus group discussions and observations. By examining the intervention’s processes, we aim to inform post-project sustainability and scaling plans. Outcomes will be assessed at baseline, three months, and at the follow up periods of six months and 12-month follow-up by trained research assistants.

### Data collection and management

To gather in-depth data on the LSE intervention, we will employ a mixed-methods Quantitative data will be collected using a combination of interviewer-administered questionnaires and self-interviewing. Questionnaires will be translated and back-translated into Hausa and pilot-tested for comprehension and cultural appropriateness. Data will be entered into a secure, password-protected database using unique participant identification numbers to maintain confidentiality.

Qualitative data from participants, program facilitators and stakeholders will be collected through facilitator logs, in-depth interviews, focus group discussions, and participant observations by the research assistants. Interviews and discussions will be audio-recorded, following consent from participants and stored in secured system, transcribed verbatim, and translated into English by external vendors, for analysis. All transcribed, anonymized and translated interviews will be reviewed by the study team for accuracy before analysis. Field notes and memos will be used to capture contextual details and researcher reflections. Data quality will be ensured through regular monitoring, double data entry, and range and consistency checks.

### Data analysis

Implementation outcomes will be assessed using the Acceptability of Intervention Measure (AIM), Intervention Appropriateness Measure (IAM), Facilitator Fidelity logs, observation checklist of program implementation and observation of staff training on the life skills curriculum, and Feasibility of Intervention Measure (FIM) [59]. Cost-effectiveness will be calculated by estimating the total program costs and comparing them to the achieved health outcomes.

Qualitative data analysis will employ a reflexive thematic analysis approach using both inductive and deductive coding strategies to identify key themes related to implementation, acceptability, fidelity, and feasibility. Transcripts will be coded iteratively to identify emerging themes and patterns, with constant comparison within and across cases. Memos and matrices will be used to facilitate synthesis and interpretation. Triangulation of qualitative and quantitative findings will be performed to enhance validity and identify convergent and divergent results [60, 61].

Quantitative data analysis will follow an intention-to-treat approach, using mixed-effects linear regression models to assess intervention effects on primary and secondary outcomes. Models will include fixed effects for time, group assignment, and their interaction, and random effects for clusters and participants. Baseline characteristics will be included as covariates to adjust for potential confounding. missing data will be handled using list wise deletion (complete case analysis) or multiple imputation (MI) methods.

Effect sizes will be expressed using Cohen’s (standardized mean differences) with 95% confidence intervals. To account for potential biases from unmeasured confounders, propensity score matching will be employed to adjust the effect size estimates.

intervals. Subgroup analyses will explore differential effects by gender, age, displacement duration, and baseline symptom severity. Sensitivity analyses will be conducted to assess the robustness of the findings to different assumptions and methods.

## Discussion

The RESETTLE-IDPs study aims to generate timely and rigorous evidence on the effectiveness and implementation of a LSE intervention for improving mental health and psychosocial outcomes among internally displaced men and women in Nigeria. The hybrid type 2 effectiveness-implementation design allows for the simultaneous evaluation of clinical outcomes and implementation strategies, with a focus on context-specific adaptation and sustainability.

The study addresses several key gaps in the current evidence base on mental health interventions for conflict-affected populations. First, it focuses on marginalized and underserved populations of IDPs in Nigeria, often excluded in MPHSS intervention design. These populations face unique challenges and vulnerabilities that require tailored and culturally relevant support. Second, it evaluates the comparative effectiveness of in-person and mobile-delivered LSE interventions, providing insights into the potential of digital technologies to expand access to mental health services in low-resource humanitarian settings. Third, it incorporates implementation science frameworks and mixed methods to assess the contextual factors, barriers, and facilitators that influence the adoption, fidelity, and sustainability of the intervention in real-world conditions.

The study also employs a community-based participatory approach, engaging IDPs, community leaders, and humanitarian actors as active partners in the design, implementation, and evaluation of the intervention. This approach aims to enhance the cultural relevance, acceptability, and ownership of the intervention, as well as build local capacity for mental health promotion and psychosocial support.

Potential limitations of the study include the reliance on self-report measures, which may be subject to social desirability and recall bias; the potential for contamination between study arms, given the close-knit nature of IDP communities; and the challenge of ensuring long-term follow-up and sustainability of the intervention in a volatile and resource-constrained setting [62]. Strategies to mitigate these limitations include the collection of detailed contact information, geographically distinct clusters, and the engagement of camp leadership, local partners and stakeholders to support the integration and scale-up of the intervention [62]. The average length of stay in IDP camps varies depending on the context, ranging from several months to several years [63]. The researchers will collect information on the length of stay in the camps as part of the baseline assessment to better understand the mobility patterns of the study population.

Despite these limitations, the RESETTLE-IDPs study has the potential to significantly contribute to the field of global mental health and humanitarian assistance. The study findings can inform the development and implementation of evidence-based, scalable, and sustainable mental health interventions for conflict-affected populations in Nigeria and other low- and middle-income countries. The study can also contribute to the growing body of research on the use of digital technologies and implementation science methods to enhance the reach, quality, and impact of mental health services in challenging contexts.

By generating rigorous, actionable evidence on LSE delivery models and implementation strategies, the RESETTLE-IDPs study will inform the development and scale-up of culturally relevant, community-based mental health interventions for conflict-affected populations. The study findings will support policymakers, practitioners, and communities in advancing comprehensive, sustainable psychosocial support and resilience-building programs that empower IDPs to thrive beyond survival. Given the growing global displacement crisis, there is an urgent need for research that illuminates effective, scalable approaches to promote the mental health and dignity of migrants and refugees in humanitarian contexts.

## Supplementary Information

Supplementary Material 1.

Supplementary Material 2.

## Data Availability

No datasets were generated or analysed during the current study.

## Appendix

### Role of committee members and team

The RESETTLE-IDPs research team assembled for this project brings a unique expertise and experience to effectively design, implement, evaluate, and sustain the proposed psychosocial intervention for internally displaced persons in Nigeria. Dr. Ejemai Eboreime, has deep expertise in implementation science, digital health innovations, and providing care for vulnerable populations including IDPs in Nigeria. As a certified project management professional (PMP), he has prior experience in leading large scale population health projects in Nigeria. Dr. Vincent Agyapong, Professor of Psychiatry and Global Mental Health, adds critical expertise in task shifting and training community health workers to provide basic mental healthcare in low resource settings like Nigeria. His research has focused on expanding access to quality mental health services through innovative approaches. Dr. Agyapong has spearheaded major initiatives to promote psychiatry and mental healthcare capacity in West Africa. Dr. Rita Orji, Canada Research Chair in Persuasive Technology, will bring her extensive research on designing interactive systems and digital health tools to motivate beneficial behaviors and reach underserved vulnerable groups. Her lab has applied these techniques to improve health behaviors including mental health. Dr. Jidda Said, Professor of Psychiatry in northeast Nigeria, will lend crucial on-the-ground clinical experience providing mental healthcare to IDPs at the epicenter of the humanitarian crisis. His insights will help tailor the program to IDPs’ realities. The local implementation partner, Brooks Insights Nigeria, led by Chisom Obi-Jeff, will coordinate key implementation tasks across sites, including participant recruitment, intervention delivery, data collection, and tracking. Brooks Insight has extensive technical expertise in research, monitoring and evaluation, capacity building, and technical assistance for health programs in Nigeria. The institution has strong networks with government, communities, and local partners that can be leveraged for stakeholder engagement, intervention delivery, data collection, and sustainability planning. Chisom Obi-Jeff has successfully managed similar donor-funded projects before, including an intervention for IDPs funded by Grand Challenges Canada. Key Nigerian knowledge users like emergency response and health officials will align the project with government priorities and facilitate real-world application. These include Lydia Wagami an assistant director of the National Emergency Management Agency (NEMA) of Nigeria. who plays a crucial role in leading National Search and Rescue operations during times of national emergencies; Dr. Bala Harri, a health policy advisor with the Federal Ministry of Health; Dr. Tunde Ojo, a psychiatrist and head of the mental health unit at the Federal Ministry of Health; Vannessa Offiong, a CNN journalist with extensive investigation experience into IDPs in Nigeria. Partnerships with local universities, NGOs and media will further strengthen outreach, coordination and impact. This stellar transdisciplinary team will draw on their combined expertise to carry out community-engaged, evidence-based research that makes a tangible difference in improving psychosocial wellbeing for conflict-affected IDPs in Nigeria. Dr. Ihoghosa Iyamu is a physician and implementation scientist with extensive research on human-centered design, implementation, and evaluation of digital interventions for public health, particularly marginalized populations. He also has experience with designing curricula and will contribute this expertise to this project.

Steering committee and data monitoring

An independent Trial Steering Committee (TSC), comprising experts in global mental health, humanitarian research, and Nigerian IDP affairs, will oversee the RESETTLE-IDAs study. Meeting bi-annually, the TSC will review progress, ensure protocol adherence, and advise on challenges. Given the low-risk nature of this behavioral intervention, the TSC will also monitor safety data, negating the need for a separate Data Safety Monitoring Board. Data quality will be maintained through regular audits by the project manager, automated validation checks in our electronic data capture system, and ongoing field staff training. The Principal Investigator will provide quarterly progress reports to the TSC, covering recruitment rates, data quality metrics, and any adverse events. An interim analysis at the study’s midpoint will assess safety and futility, without formal stopping rules for efficacy. This pragmatic approach balances rigorous oversight with the real-world context of IDP settings, ensuring participant safety and data integrity while maintaining operational flexibility necessary for humanitarian research.

## Abbreviations

GBV: Gender-based violence
LSE: Life Skills Education
IDPs: Internally displaced persons
RESETTLE-IDPs: Rebuilding Emotional Stability and Strength Through Therapeutic and Life-Skills Education for Internally Displaced Persons in Nigeria
UNICEF: United Nations Children’s Fund
RE-AIM framework: Reach, Effectiveness, Adoption, Implementation, Maintenance framework
